# Hypoxia as a Modulator of Inflammation and Immune Response in Cancer

**DOI:** 10.3390/cancers14092291

**Published:** 2022-05-04

**Authors:** Rosa A. Castillo-Rodríguez, Cristina Trejo-Solís, Alfredo Cabrera-Cano, Saúl Gómez-Manzo, Víctor Manuel Dávila-Borja

**Affiliations:** 1 Laboratorio de Oncología Experimental, Instituto Nacional de Pediatría, Mexico City 04530, Mexico; alfredocc@xanum.uam.mx; 2 Programa Investigadoras e Investigadores por México, Consejo Nacional de Ciencia y Tecnología (CONACYT), Mexico City 03940, Mexico; 3 Laboratorio Experimental de Enfermedades Neurodegenerativas, Instituto Nacional de Neurología y Neurocirugía, Mexico City 14269, Mexico; cristina.trejo@innn.edu.mx; 4 Posgrado en Biología Experimental, División de Ciencias Biológicas y de la Salud, Universidad Autónoma Metropolitana-Iztapalapa, Mexico City 09340, Mexico; 5 Laboratorio de Bioquímica Genética, Instituto Nacional de Pediatría, Mexico City 04530, Mexico; saulmanzo@ciencias.unam.mx; 6 Independent Researcher, Dallas, TX 75149, USA; victorm.davila.borja@gmail.com

**Keywords:** hypoxia, inflammation, cancer, tumoral microenvironment, HIF-1α, NF-κB, STAT

## Abstract

**Simple Summary:**

The tumoral microenvironment comprises cancer cells and surrounding components, including immune and endothelial cells, along with the extracellular matrix. As the tumoral cells proliferate, a gradient of oxygen and nutrients is established while the tumor becomes a solid mass. Tumoral cells have developed strategies to adapt themselves to the hypoxic microenvironment and to modify the tumoral microenvironment, including the inflammatory cells, in order to maintain their proliferation and ulterior metastasis, representing a crucial factor in the malignity of the disease. Therefore, we analyze the signaling and cellular components that interconnect inflammation and hypoxia, emphasizing the most recent findings and contributing to their understanding, as a reference for new therapeutic strategies.

**Abstract:**

A clear association between hypoxia and cancer has heretofore been established; however, it has not been completely developed. In this sense, the understanding of the tumoral microenvironment is critical to dissect the complexity of cancer, including the reduction in oxygen distribution inside the tumoral mass, defined as tumoral hypoxia. Moreover, hypoxia not only influences the tumoral cells but also the surrounding cells, including those related to the inflammatory processes. In this review, we analyze the participation of HIF, NF-κB, and STAT signaling pathways as the main components that interconnect hypoxia and immune response and how they modulate tumoral growth. In addition, we closely examine the participation of the immune cells and how they are affected by hypoxia, the effects of the progression of cancer, and some innovative applications that take advantage of this knowledge, to suggest potential therapies. Therefore, we contribute to the understanding of the complexity of cancer to propose innovative therapeutic strategies in the future.

## 1. Introduction: Hypoxia and Inflammation as a Cancer Hallmark

Chronic inflammation and viral infections often precede the development of cancer. In fact, the association between cancer and inflammation has been reported frequently; as examples, we can mention ulcerative colitis and colorectal cancer; hepatitis B or C and hepatocellular carcinoma (HCC); tobacco consumption and lung cancer; and human papillomavirus infection and cervical cancer, among many other cases [1,2,3,4]. Therefore, a clear connection has been established, implicating that inflammation as immune response promotes oncogenic transformation or supports tumoral progression. Moreover, the cells that coordinate the inflammatory response are responsible for the recognition and elimination of tumoral cells, which develop mechanisms to avoid these processes. 

In this complex context, tumoral hypoxia plays an important role in the regulation of the metabolism and the elements that integrate the tumoral microenvironment (TME). The cellular components of the TME include cancer cells as well as immune cells like lymphocytes T and B, tumor-associated macrophages (TAMs), cancer-associated fibroblasts (CAFs), natural killer (NK) cells, Myeloid-Derived Suppressor Cells (MDSCs), tumor-associated neutrophils (TANs), dendritic cells, mast cells, granulocytes as well as adipocytes, endothelial cells and pericytes [5,6]. An intricate communication is established between cancer and its surrounding cells, releasing several cytokines and molecules to modulate the environment and allow tumoral growth and metastasis. Furthermore, tumoral progression requires extra support of oxygen and energy. In these circumstances, the tumor develops hypoxia, a stressful factor that modifies the phenotype of the cancerous and surrounding cells in order to allow their adaptation and survival. The interconnection between hypoxia and inflammation has been recognized and plays different roles at several levels of the progression of cancer. 

Here, we analyze the effect of tumoral hypoxia over the inflammatory response, including the signaling pathways that link the hypoxic and inflammatory process, and then analyze their particularities in the immune cellular components. Finally, we analyze some future therapeutic approaches that have been developed as a result of the knowledge in this field. 

## 2. Regulation of Inflammatory Pathways by Hypoxia

Hypoxia is a factor that awakens a series of signaling responses in cells in order to adapt their cellular processes to the lack of oxygen, like metabolism. However, hypoxic mediators interact with other important pathways that influence the immune response or vice versa. We consider the participation of HIF, nuclear factor kappa B (NF-κB), and Signal transducer and activator of transcription (STAT) pathways as the main mediators of the inflammatory response and hypoxia, and therefore we analyze them hereafter.

### 2.1. HIF Pathway

The understanding of how cells adapt to low levels of oxygen is considered crucial. In fact, the 2019 Nobel Prize in Physiology or Medicine was awarded to William Kaelin, Peter Ratcliffe, and Gregg Semenza for their studies in cellular oxygen sensing and adaptability [7]; furthermore, the hypoxia inducible factors (HIFs) are the key point in this phenomenon. 

The HIFs belong to a transcription factor family characterized by a basic helix-loop-helix (bHLH)/PAS domains, which regulate the cell adaptation to the hypoxic microenvironment [8,9,10]. This family contains three α isoforms, known as HIF-1α, HIF-2α, also called endothelial PAS domain-containing protein 1 (EPAS1), and HIF-3α. Another member of the HIF family is the β isoform (HIF-1β), also called aryl hydrocarbon receptor nuclear translocator (ARNT) [8,10,11,12].

HIF-1α and HIF-2α have 48% identity in their amino acid sequence; in fact, the maximal conservation regions are in the bHLH (85%), PAS-A (68%), and PAS-B (73%) domains. Both isoforms also share sequence identity in the C-terminal domain, which possesses a hypoxia response domain [12]. HIF-3α shares high similarity to HIF-1α and HIF-2α in the bHLH and PAS domains; however, HIF-3α does not have a transactivation domain, like HIF-1α and HIF-2α [13]. The α subunits bind with the β subunits to form active heterodimers that bind to DNA. The most studied heterodimer is HIF-1α/HIF-1β [8,10,12,13]. 

**Figure 1:**
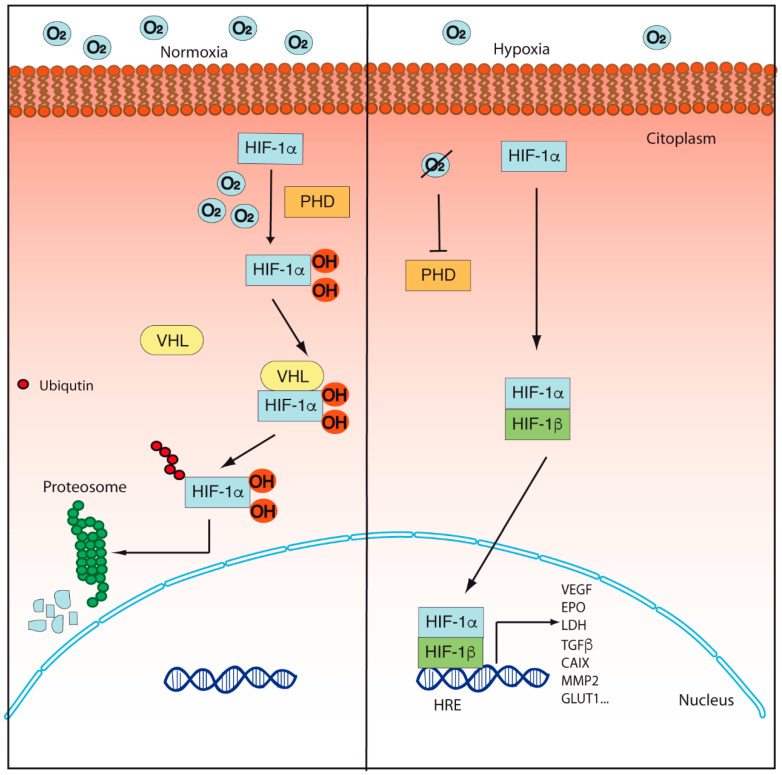
HIF-1α pathway. In normoxia (**left**), HIF-1α is hydroxylated by PHDs to be recognized by VHL, which adds ubiquitin units to send HIF-1α to the proteosome to be degraded. In hypoxia (**right**), PHDs are inactivated and HIF-1α is translocated to the nucleus, forming a dimmer with HIF-1β to induce the transcription of several target genes. VEGF, Vascular Endothelial Growth Factor; EPO, erythropoietin; LDH, Lactate Dehydrogenase; TGF-β, Transforming Growth Factor β; CAIX, Carbonic Anhydrase 9; MMP2, Matrix Metallopeptidase 2; GLUT1, Glucose transporter 1.

Under normoxic conditions, the HIF-α subunits are regulated by post-translational modifications that allow their degradation. These modifications are mainly hydroxylations in proline residues, which are carried out by the activity of enzymes called prolyl hydroxylases, which use oxygen as a substrate (Figure 1) [14]. In mammals, three isoforms of prolyl hydroxylases (PHD) have been identified: PHD1, PHD2, and PHD3 [14]. However, it has been reported that PHD2 is the most important in the hydroxylation and degradation of HIF-1α and HIF-2α, since the inhibition of the expression of PHD2 supports angiogenesis and promotes the stability and transcriptional activity of HIF-1α [15]. For instance, Hsu and colleagues demonstrated that lung cancer cells secrete an exosomal microRNA called mir23-a, which binds to the 3 ‘UTR region of PHD2, thus inhibiting its expression and promoting the transcriptional activity of HIF-1α, angiogenesis, and tumoral progression [16]. 

The hydroxylation in proline 564 is recognized by the tumor suppressing protein von Hippel-Lindau (pVHL), which acts as an E3 ubiquitin ligase by adding ubiquitin residues to the HIF-α subunits, thus promoting their degradation (Figure 1) [17,18,19,20]. Loss of function or inhibition of pVHL expression prevents HIF-1α degradation, which supports tumor progression. For instance, Chakraborty and colleagues demonstrated that epigenetic regulation, specifically methylations in the VHL promoter, decreases its expression and thus increases the levels of HIF-1α and vascular endothelial growth factor (VEGF) [21]. Besides PHD, other hydroxylations are carried out on asparagine residues by another enzyme known as factor inhibiting HIF (FIH) [22,23]. Hydroxylations at asparagine residues prevent HIF from binding to transcriptional cofactors such as p300, thus inhibiting its activity [22,23,24].

Under hypoxia, the activity of PHD enzymes is compromised, and HIF-α subunits are not degraded; this allows cellular adaptation to hypoxic stress (Figure 1) [25,26]. HIF-1α and HIF-2α differently regulate the cellular response to hypoxia. Holmquist-Mengelbier and colleagues suggested that HIF-1α is the main regulator of the response to acute hypoxia, while HIF-2α is the main regulator of the response to chronic hypoxia, using an in vitro model of neuroblastoma [26]. 

After the stabilization of HIF-α subunits, they are translocated to the nucleus and form a heterodimer with the HIF-1β subunit, which binds to DNA sequences called hypoxia response elements (HREs) [8,11]. This union activates the transcription of different genes related to mesenchymal-epithelial transition, metastasis, and angiogenesis, such as erythropoietin, VEGF, among others, but also genes related to metabolism and biotransformation as glucose transporter (GLUT) 1, hexokinase 2 (HK2), lactate dehydrogenase A (LDHA), carbonic anhydrase IX (CAIX), enolase 1 (ENO1), solute carrier family 2 member 2 (SLC2A1), and even cytochromes P450 [11,27,28,29,30,31,32,33,34,35]. In this sense, Valencia and colleagues, using medulloblastoma cells, demonstrated that hypoxia and HIF-1α promote resistance to cyclophosphamide and ifosfamide by decreasing the expression of cytochromes P450 CYP2B6, CYP3A4, and CYP3A5 [36]. Recently, in our laboratory, we demonstrated that hypoxia increased the expression of the isoforms CYP24A1 and CYP2S1 related to the activity of HIF-1α in a liver cancer cell model [32]. 

Interestingly, other alternative signaling pathways can promote the degradation and inhibition or the stabilization and activation of HIF-α subunits. For instance, Bao and colleagues demonstrated that G9a and G9a-like protein (GLP) are methyltransferases that methylate HIF-1α in its transactivation domain, specifically in lysine 674, thus inhibiting its transcriptional activity. They also demonstrated that these enzymes only methylate HIF-1α and not HIF-2α [30]. Moreover, the heat shock protein 90 (Hsp90) binds to HIF-1α, thus supporting its stabilization [35]. Another signaling pathway that positively participates in the transcription and translation of HIF-α is the pathway regulated by PI3K/AKT/mTOR proteins [33]. In this sense, the genetic and pharmacological inhibition of PI3K/mTOR signaling induces a reduction of tumor hypoxia [37]. Phosphorylation is another mechanism that increases HIF activity. For example, the phosphorylation of HIF-1α at serine residues 641 and 643 by nuclear protein kinases such as p42/p44 MAPK improves its transcriptional activity and its accumulation in the nucleus [38]. Likewise, the phosphorylation of HIF-2α at the serine residue 672 by ERK1/2 results in its transcriptional activity and nuclear accumulation [39]. 

In recent years, several studies have shown the role of long non-coding RNAs in cancer progression [40,41]. Wen and colleagues demonstrated that the long non-coding RNA DANCR promotes the stability of HIF-1α mRNA, supporting cancer cell invasion and migration [40]. Barth and colleagues reported the role of other long non-coding RNAs in the regulation of HIF-α [41].

**Figure 2:**
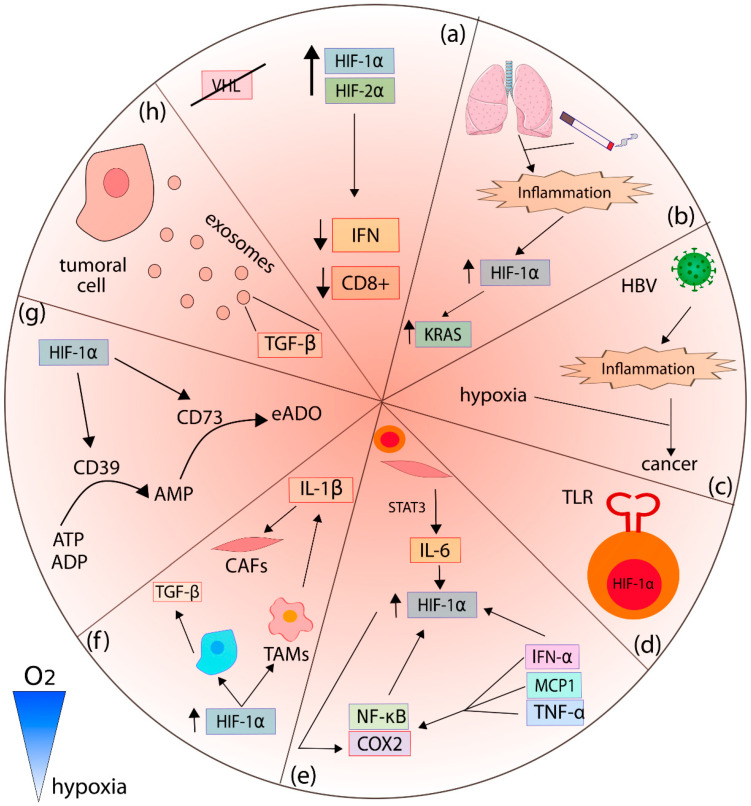
Interrelation between the HIF pathway, inflammation, and cancer. (**a**) In ccRCC, VHL is inactive, leading to HIF-1α and HIF-2α accumulation. As a consequence, there is a decrease in antitumoral response due to low levels of HIF-1α, IFN, and CD8+ inactivated cells. (**b**) Inflammation- induced by COPD with the overexpression of HIF-1α leads to overactivation of KRAS signaling and cancer. (**c**) Viruses such as HBV also induce inflammation that synergizes with hypoxia as factors to induce cancer. (**d**) Correlation between the expression of TLR and nuclear HIF-1α was observed in early carcinogenesis of the pancreas. (**e**) IL-6, NF-κB, and IFN-α induce the overexpression of HIF-1α; indirectly, TNF-α and MCP1 also induce HIF-1α through the NF-κB/COX2 axis. HIF-1α also induces the expression of COX2. (**f**) HIF-1α stimulates TAMs and tumoral cells to release IL-1β, which stimulates CAFs. Tumoral cells also secrete TGF-β. (**g**) HIF-1α induces and regulates the expression of CD39 and CD73 to obtain eADO. (**h**) Hypoxic tumoral cells release exosomes enriched with molecules, such as TGFβ-inducing M2 TAMs recruitment. Abbreviations: clear cell renal cell carcinoma, ccRCC; chronic obstructive pulmonary disease, COPD; hepatitis B virus, HBV; toll-like receptors, TLR; interleukin 1-beta, IL-1β; tumor necrosis factor-α, TNF-α; interferons, IFN; monocyte chemoattractant protein 1, MCP1; extracellular adenosine, eADO. See more details in the main text. Lung icon of Figure 2b is from Servier Medical Art. Servier Medical Art by Servier is licensed under a Creative Commons Attribution 3.0 Unported License (https://creativecommons.org/licenses/by/3.0/, accessed on 2 January 2022).

The interrelation between the HIF pathway, inflammation, and cancer has been extensively described, finding examples in several cancer types (Figure 2). Clear cell renal cell carcinoma (ccRCC) is particularly HIF-dependent; it is characterized by an accumulation of HIF-1α and HIF-2α due to a mutational inactivation of VHL, independently of the oxygen levels. Using human ccRCC cell lines, it has been reported that HIF-1α is essential for tumor formation and induces a glycolytic profile, while HIF-2α regulates lipoprotein metabolism, biogenesis of ribosome, and induction of MYC and E2F. More importantly, the proteomic analysis showed that HIF-2α-deficient tumors presented a better anti-tumoral response, demonstrated by an increment in the antigen presentation, interferon (IFN) signaling, and CD8+ T cell infiltration and activation. This would suggest that HIF-2α is also a repressor of the anti-tumoral immune response, even independently of hypoxia or HIF-1α [42] (Figure 2a). Interestingly, HIF-2α induces the expression of the Programmed death-ligand 1 (PD-L1), as observed in ccRCC [43]. PD-L1 binds to its transmembrane receptor, the programmed cell death protein 1 (PD-1), which is usually expressed in cytotoxic T lymphocytes, suppressing their activation and thus the immune response. Thus, immunotherapy with HIF-2α inhibitors should consider that PD-L1 expression could also be reduced and might reduce the effectivity of the immunotherapy [44]. Moreover, VHL disease can lead to the development of highly vascularized tumors in addition to ccRCC, such as hemangioblastomas in the brain or retina, and pheochromocytoma. The constant activation of the HIF pathway leads to the expression of proangiogenic proteins such as VEGF and other HIF targets related to the progression of the tumor and inflammation [45]. However, *VHL* mutations could be related to alterations in other pathways independent of HIF, including those related to inflammation. In this respect, the downregulation of Endoglin (ENG) and tumor necrosis factor (TNF) α, which is an activator of the NF-κB pathway, has been reported. As expected, a decrease in the expression of targets of NF-κB as matrix metalloproteinases (MMPs), cyclooxygenase 2 (COX2), and nitric oxide synthase (NOS)3 were revealed by RNAseq of blood outgrowth endothelial cells from VHL disease patients, as a model to assess the systemic effects in other organs predisposed to develop malignant tumors [46].

Smoking is the principal inductor of an inflammatory process known as chronic obstructive pulmonary disease (COPD), which is considered a risk factor to develop lung cancer; moreover, the inflammation derived from COPD and the overexpression of HIF-1α potentiate the activation of KRAS signaling, which in turn induce tumorigenesis. In fact, the deficiency of HIF-1α decreases epithelial inflammation and avoids the induction of lung cancer, even in the presence or absence of COPD, in a murine model [47] (Figure 2b).

The oxygen levels and expression of HIF-1α could be also regulated by microbiota linked to chronic inflammation [1]. In the intestinal microenvironment, some microorganisms release peptides that induce an inflammatory response, which has been related to chronic inflammation and cancer [48,49]. But in other cases, the microbiota could exert a protective effect in cancer and also be synergistic to cancer treatment as an immunomodulator [50]. Reciprocally, hypoxia could also modulate the presence of specific microbiota, which is associated with chronic inflammation and the presence of colorectal or liver cancer [51].

In HCC, the most frequent form of liver cancer, a correlation has been established between inflammation, hypoxia, and cancer; however, the participation of hepatitis B virus (HBV) as an inductor of the HIF pathway has been questioned. In this sense, an in vivo model using mice infected with HBV to produce chronic hepatitis B as a precursor of HCC was used to evaluate their association with hypoxia. The authors found an increase in the hypoxic profile genes of chronic hepatitis B groups without cirrhosis or HCC but no evidence that regulatory hepatitis B X protein (HBx) could modulate the HIF expression, contrary to previous reports. However, independently of the HBx participation, a clear connection between inflammation and hypoxia activation pathways was confirmed as a potent precursor of HCC (Figure 2c) [52].

In a study of pancreatic cancer samples, the expression of toll-like receptors (TLRs), a characteristic type of single-pass membrane-spanning receptor expressed in immune cells, was associated with HIF-1α and CAIX. Particularly, TLR2 and TLR9 showed a correlation with nuclear HIF-1α only in early pancreatic lesions as pancreatic intraepithelial neoplasia type I and II, but not in type III or pancreatic carcinoma, which implies that innate inflammation and hypoxia are coexistent factors that could be involved in early carcinogenesis (Figure 2d) [53].

Furthermore, TLR expression has been abnormally found in several types of tumoral cells, such as endometrial, oral, glioma, prostate, liver, endometrial, gastric, and pancreas [53,54,55,56,57,58,59]. It has been reported that TLR3 could induce the expression of the I.3 isoform of HIF-1α, which accumulates in the nucleus even in normoxia, promoting the secretion of VEGF and tumor progression in prostate cancer [56]. In glioma biopsies, TLR4 was overexpressed. Moreover, TNF-α induced the activation of the TLR4-AKT-HIF-1α axis [55]. A feedback mechanism was found in oral squamous cell carcinoma (OSCC), where the activation of TL3 and TLR4 induced the expression of HIF-1α; in addition, HIF-1α bound to the promoter of TLR3 and TLR4, increasing their expression [54]. Furthermore, it has been documented that hypoxia induces the overexpression of TLR9 in HCC. Hypoxia promotes the translocation from the nucleus to the cytoplasm of damage-associated molecular pattern (DAMP) proteins, such as high mobility group box 1 (HMGB1) and mitochondrial DNA (mtDNA). Then, HMGB1 and mtDNA can activate endosomal TLR9, leading to tumor progression in a feedback mechanism [57]. In endothelial cells, Biglycan (BGN), a protein from the extracellular matrix, interacts with TLR2 and TLR4, leading to an increase in the HIF-1α activity, including VEGF overexpression, which in turn promotes gastric cancer progression and metastasis [59].

The correlation between HIF and several cytokines, such as interleukin (IL)-1β, IL-6, and IFN, has also been reported. For example, IL-6 is a pleiotropic cytokine synthesized by immune cells, endothelial cells, fibroblasts, myocytes, and adipocytes; moreover, IL-6 modulates inflammation and enhances the transcription and nuclear translocation of HIF-1α through the STAT3 pathway [60]. The expression of HIF-1α is also induced by IFN-α, a cytokine released by lymphocytes in response to viral infections, apparently through the Janus Kinases (JAK)/PI3K/mTORC2 signaling pathway [61]. Piasecka and colleagues demonstrated that some cytokines such as IL-1β, TNF-α, IFN, and monocyte chemoattractant protein 1 (MCP1) stimulate the NF-κB/COX2 signaling pathway, which induces the overexpression of HIF-1α and HIF-3α in tumoral cells and mesenchymal stem cells [62,63]. HIF-1α also leads to the expression of COX2, an enzyme that metabolizes the synthesis of prostaglandins [64,65] (Figure 2e). On the contrary, HIF-1α could induce the secretion of other cytokines, such as IL-1β by TAMs and cancer cells stimulated by hypoxia [65,66]. For instance, breast cancer cells that were stimulated by hypoxia released IL-1β, which in turn induced the activation of invasive CAFs (Figure 2f) [65].

A clear association between hypoxia and the induction and secretion of the transforming growth factor (TGF)-β has been established in several types of cancer, such as pancreatic, colorectal, kidney, lung, melanoma, oral, and breast [67,68,69,70,71,72,73,74,75,76,77]. TGF-β is a pleiotropic cytokine linked to the regulation of the epithelial-mesenchymal transition (EMT) related to metastasis, suppression of the immune response, and regulation of TME cellular components such as cancer stem cells (CSCs), CAFs, and TAMs [69,71,73,75,78,79].

In particular, TGF-β inhibits glycolysis in normoxia; however, this is reverted in hypoxia. Using a lung cancer model, it has been reported that the TGF–β/Smad signaling pathway leads to the phosphorylation of Smad2 and Smad3; in hypoxia, HIF-1α binds to Smad3 phosphorylated, inducing the expression of c-Myc, which induces the expression of protein machinery to alternative splicing targeting PKM2, which in turn activates glycolysis [74].

Interestingly, a decrease in the activation of the TGF-β pathway through the downregulation of the TGF-β receptor type 2 (TGFBR2) has been associated with hypoxia. Apparently, hypoxia induced the expression of the enhancer of zeste homolog 2 (EZH2), a histone methyltransferase, which in turn regulates the hypermethylation of the TGFBR2 promoter in a model of prostate cancer [80]. In another approach, when TGFBR2 is depleted in CD4+ T cells, a remodeling of the vasculature in the tumor leads to the death of tumoral avascular regions and suppresses the tumor progression [79].

A mechanism of resistance of immunotherapy has been related to the influence of HIF-1α over the extracellular adenosine (eADO) axis. ATP/ADP in the extracellular space is converted to adenosine monophosphate (AMP) by the ectonucleotidase CD39; then, AMP is metabolized by CD73 to produce eADO, which has multiple immunosuppressive functions through its interaction with adenosine receptors (ARs) such as A1, A2A, A2B, and A3. Interestingly, HIF-1α induces and regulates the expression of ectonucleotidases CD39 and CD73, along with A2A and A2B receptors, in tumors (Figure 2g) [81,82,83,84,85].

Tumoral cells release exosomes that contain diverse elements, allowing their communication with near or distant cells distributed through the body. Interestingly, a hypoxic environment increases the number of extracellular vesicles in some types of cancer, such as breast or lung. In addition, it has been found that pancreatic cancer cells release exosomes enriched with miR-301a-3p after a hypoxic stimulus, targeting surrounding macrophages and inducing their transition to an M2 pro-tumoral phenotype, which facilitates the progression of cancer [86]. Likewise, another group described that hypoxic tumoral cells release exosomes enriched with chemokines/chemoattractants, macrophage colony-stimulating factor (M-CSF), monocyte chemoattractant protein-1(MCP1), C–C motif chemokine (CCL) 2, endothelial monocyte-activating polypeptide II (EMAP II), leukotriene A-4 hydrolase (LTA4H), TGF-β1, TGF-β2, TGF-β3, macrophage migration inhibitory factor (MIF), and ferritin heavy/light chain (FTH, FTL). These chemokines promote recruitment and M2 macrophage polarization (Figure 2h) [87].

The role of cancer stem cells (CSCs) as initiators of a cancerous population has also been discussed, and the hypoxic environment seems a key factor in CSC activation. For example, it has been reported that CCAAT-enhancer-binding proteins (C/EBP) δ induce a CSC phenotype and interestingly, hypoxia and IL-6 enhanced their expression. Moreover, there is a feedback loop that allows C/EBPδ to amplify IL-6 and HIF-1α expression, as was observed in a breast cancer model [88].

As mentioned above, in our laboratory, we report that hypoxia promotes the overexpression of cytochrome P450 CYP2S1 [32]. This enzyme is important, since it participates in the regulation of inflammatory molecules such as arachidonic acid, linoleic acid, prostaglandins, and thromboxane [89]. However, the role of CYP2S1 in inflammation, carcinogenic processes, and tumor hypoxia remains unclear [90].

We will describe with more detail the interrelation between hypoxia and some of the most important inflammatory pathways, such as NF-κB and STAT3, in the next sections.

### 2.2. NF-κB Pathway

It has been documented that the correlation between the activation of the NF-κB pathway and hypoxic conditions, particularly linked to HIF, is the principal mediator in the hypoxic response facilitating the progression of cancer [91,92]. Usually, the NF-κB pathway is related to the response to inflammation mediated by the immune system. However, it is also implicated in other important physiological functions, such as apoptosis evasion, proliferation, as well as cell adhesion and tissue remodeling. Consequently, NF-κB deregulation has been involved in many human diseases, including cancer [93,94].

**Figure 3:**
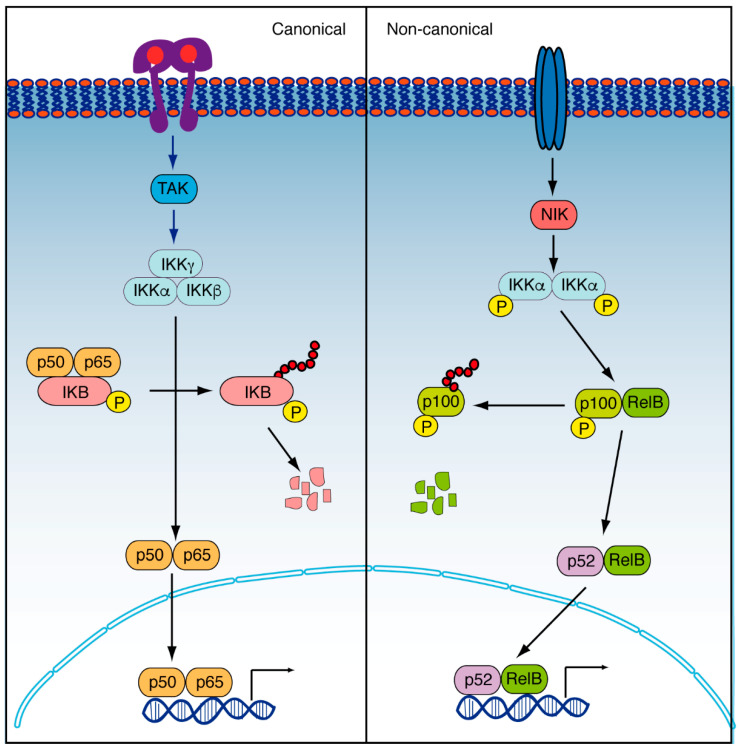
Canonical and non-canonical pathway of NF-κB. On the (**left**), p50/p65 subunits are inhibited by IκBs; however, when the pathway is activated, IκBs are phosphorylated by the IKK complex integrated by IKKα, IKKβ, and IKKγ and sent to degradation. Then, the p50/p65 are released and translocated in the nucleus to activate the transcription of the target genes. On the (**right**), in the non-canonical pathway, RelB is sequestered by p100. When the pathway is activated, IKKα phosphorylates and sends p100 to degradation. Then, RelB is released and forms a dimer with p52, which is translocated to the nucleus. Abbreviations: IκB kinase complex (IKK); Transforming growth factor-β–activated kinase 1 (TAK); NF-κB-Inducing Kinase (NIK).

The NF-κB complex is a family of nuclear transcription factors that recognize a consensus DNA sequence (GGGRNYYYCC, where R is a purine, Y is a pyrimidine, and N is any nucleotide) and mediate the induction or inhibition of the transcription of their target genes [95,96]. This family includes RelA (p65), RelB, c-Rel, p50/p105 (NF-κB1), and p52/p100 (NF-κB2), and they are capable of forming homo- and heterodimers. They are also characterized by an N-terminal REL homology domain [97], which allows their binding to DNA and their dimerization. When this pathway is inactive, the NF-κBs are sequestered in the cytosol by the inhibitory proteins of κB (IκBs) (Figure 3). But when the pathway is active, the IκBs are phosphorylated by the IκB kinase (IKK) complex. This complex is integrated by the catalytic subunits IKKα and IKKβ and several copies of the regulatory subunit NF-κB essential modifier or NEMO, also known as IKKγ. The phosphorylation of the κBs leads to their ubiquitination and degradation, releasing the NF-κBs to be translocated to the nucleus [94]. Regularly, the activation of the pathway is derived due to the kinase activation in response to extracellular signals, including cytokines such as TNF-α, IL-1, IL-6, reactive oxygen species (ROS), or prostaglandins [98]. Alternatively, the non-canonical pathway relays in p100, which inhibits RelB. The activation of the pathway through the TNF receptor family members triggers NF-κB-Inducing Kinase (NIK), which activates IKKα to phosphorylate p100 and send it to ubiquitination. Thus, RelB is released and interacts with p52, to form a dimer that translocates to the nucleus and activates the gene transcription [99] (Figure 3).

The interaction between hypoxia and the NF-κB pathway seems to be synergistic and is highly related to HIF. In this context, abundant evidence has been reported about the crosstalk between the transcription factors NF-κB and HIF. For example, the stimulation of the NF-κB pathway by their respective ligands as TNF-α increased the HIF-1α levels, even in normoxic conditions, and activates target genes linked to hypoxic conditions. Moreover, the HIF-1α promoter has NF-κB binding sites [100]. In addition, it has been documented that hypoxia itself stimulates the NF-κB pathway through different mechanisms, which are regulated in a feedback sense. In an in vitro model with an exposition of 0.02% of O_2_, the phosphorylation and degradation of the inhibitory subunit IκBα allowed the translocation of NF-κB factors and thus the activation of the pathway [101]. In addition, it has been reported that the promoter of IKKβ has a binding site for HIF-1α; thus, IKKβ expression was induced, leading to an increase of IκBα phosphorylation and p65 activation [102]. It has been suggested that HIF regulates the TLR/NF-κB signaling pathway under hypoxic stress through the positive regulation of TLR4 transcription [54,103]. These observations confirm the correlation between HIF-1α and NF-κB pathways but also show the reliance on the activators of these pathways.

Recently, the regulation of hypoxia over NF-κB through TGF-β–activated kinase 1 (TAK) was reported [104]. This kinase mediates NF-κB activation after stimulation with the IL-1 receptor or TNF receptor. After TAK activation, it is recruited to TNF receptor–associated factor (TRAF) 6 or TRAF2. TRAF6 catalyzes the poly-ubiquitination in the lysine 63, which activates the TAK kinase complex (including TAB1, TAB2, and TAB3) and phosphorylates serine residues of IKKβ, therefore activating the NF-κB pathway [105]. Hypoxia induces the IKK activity through calcium/calmodulin-dependent kinase 2 (CaMK2). This mechanism implies Ca^2+^ release and requires the activation of TAK1. Interestingly, the inhibition of IκBα results from the SUMOylation in critical lysines and the release of RelA to activate NF-κB [106].

Furthermore, hypoxia can directly stimulate the NF-κB pathway through the inhibition of prolyl-hydroxylases, which can interact with these transcription factors. The PHDs and FIH can sense oxygen levels and catalyze the hydroxylation of the proline residues within consensus LxxLAP motifs (PHD1, PHD2, PHD3) and asparagine residues (FIH) in the target protein, as discussed earlier [107]. It has been documented that hypoxia activates IKKβ through phosphorylation dependence and consequent phosphorylation and degradation of IκBα. This interaction is regulated by the recognition of a LxxLAP consensus motif contained in IKKα/β by PHD1; as hypoxia inactivates the PHD1 hydroxylation over IKKβ, then leads to the activation of NF-κB [108], which could benefit the progression of cancer. The downregulation of PHD2 also has been correlated with NF-κB activation and inflammation. It has been reported that the estrogen receptor β (ERβ) induces the expression of PHD2, leading to a degradation of HIF-1α [109]. In contrast, chronic inflammation of the prostate is related to loss of Erβ, and the authors proposed a model in which the loss of ERβ and PHD2 downregulation, as well as hypoxia, stabilizes HIF-1α and activates inflammation through NF-κB [102]. However, other groups proposed that the pro- or anti-inflammatory effects of PHD1 and PHD2 as well as PHD expression were linked to NF-κB activation. Li and colleagues showed that, independently of HIF-1α, PHD2 controls the NF-κB/p65 transactivation in a model of nucleus pulposus in vitro with a pro-inflammatory effect; in addition, PHD2 expression seems to be regulated by NF-κB [110]. Importantly, this study suggests that NF-κB and PHD2 constitute a functional circuit, each regulating the activity of the other. Likewise, Ullah and colleagues reported that PHD1 inhibition reduced the inflammatory response in vivo but increased p53 activity [111].

PHD3 interacts with IKKβ, blocking the interaction of IKKβ and Hsp90, avoiding IKKβ phosphorylation independently of PHD3 hydroxylase activity. The low expression of PHD3 in colon cancer is related to a malignant course of the disease together with an increase of NF-κB activity [112]. In other reports, PHD3 binds to IKKγ and inhibits its ubiquitination, decreasing the NF-κB activity [113], which could be protective in the case of cancer. In fact, several reports have demonstrated that IKKγ polyubiquitination is necessary in the activation of IKKγ/NF-κB signaling [114,115,116].

On the other hand, FIH could hydroxylate proteins with an ankyrin repeat domain, including IκBα, IκBε, and p105 [117]; however, a functional effect has not yet been detected [118].

### 2.3. STAT Pathway

Another important group of proteins related to inflammation and cancer is a protein family known as signal transducer and activator of transcription proteins (STATs). STATs are cytoplasmic proteins that, after their activation, form dimers and translocate to the nucleus to induce the transcription of several genes related to proliferation or immune response [119]. STATs can be activated after the binding of certain cytokines, such as IL-6, INF-γ, or growth factors such as epidermal growth factor (EGF), to their respective receptors.

**Figure 4:**
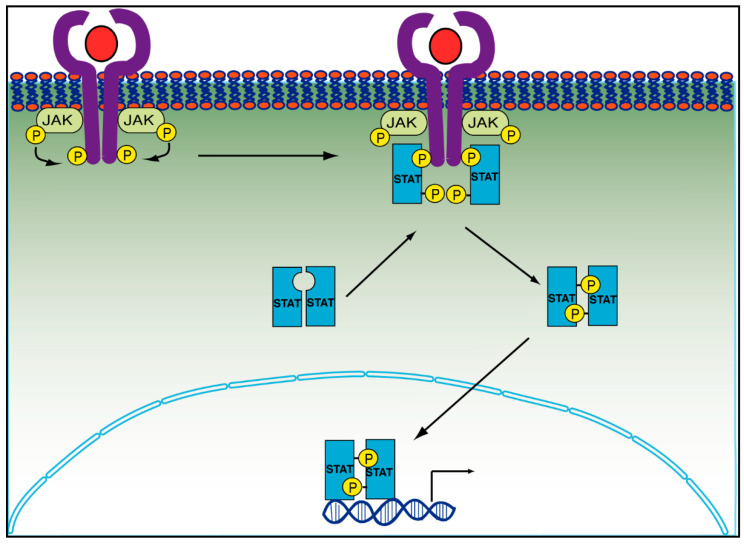
STAT pathway. After the interaction of the ligands with their corresponding receptors, associated tyrosine kinases, such as JAKs, are transphosphorylated to then phosphorylate the cytoplasmic tail receptors. Then, STATs are recruited, and both are phosphorylated to form dimers that are translocated into the nucleus to activate gene transcription (see details in the text).

After the binding of IFN-γ and other cytokines to their respective receptors, some associated-receptor tyrosine kinases, such as the JAK family, are activated [120]. In the case of JAK, the dimerization or oligomerization of the receptor allows its activation and subsequent transphosphorylation in the tyrosine residues, and in consequence activates the phosphorylation of the cytoplasmic tails of the receptors [119,121]. This allows the recruitment of STAT proteins through their Src-homology 2 domain (SH2). A third phosphorylation occurs in the tyrosine residues of STATs, which now can form homodimers (STAT1, STAT3, STAT4, STAT5aA, STAT5B) or heterodimers (STAT1 and STAT2, or STAT1 and STAT3) and are allowed to enter the nucleus to work with other co-activators of transcription factors and thus increase the transcription of several genes related to proliferation or immune response (Figure 4) [122].

Alternatively, STATs could be activated by receptors with intrinsic tyrosine kinase activity (RTKs), which bind to growth factors such as EGF or platelet-derived growth factor (PDGF). The RTKs could then activate STATs directly or indirectly through non-receptor tyrosine kinases (NRTKs), such as SRC family kinases, which are recruited to phosphorylate and activate STAT proteins. NRTK could also activate STATs even without the activation of a receptor [119,123].

In cancer, several alterations of this pathway have been reported, including mutations in JAK or STAT, overactivation by an excess of ILs, or the reduction of the negative regulators of the pathway [124]. In these circumstances, the activation of the pathway occurs in both tumoral and immune cells of the surrounding microenvironment and affects processes such as proliferation, escape of apoptosis, and metabolic adaptation. In this sense, STAT3 and STAT5 increase the expression of cyclin D2 and D1, respectively, while STAT3 downregulates p21, both increasing the cell cycle [125]. STAT3 and STAT5 also induce transcription of BCL-xL to avoid apoptosis [125,126,127]. In fact, STAT3 activation promotes the escape of immunosurveillance [128,129], contrary to STAT1, which exerts an antitumoral effect.

In particular, STAT3 has been identified as a regulator of inflammation and cancer [130]. In relation with metabolism and hypoxia, STAT3 decreases the mitochondria activity, increases glycolysis, and upregulates HIF-1α and VEGF [131,132,133,134]. Mechanistically, it has been reported that STAT3 seems to stabilize HIF-1α and prevents its degradation, but also accelerates its synthesis de novo [134]. In this sense, in has been reported that STAT3 competes against VHL in the binding of HIF-1α, inhibiting VHL activity and thus increasing HIF-1α stabilization [135]. STAT3 also interacts with HIF-1α to recruit coactivators such as CREB binding protein (CBP), p300, and RNA polymerase II (Pol II) to target gene promoters usually induced by HIF-1α activity [136].

Interestingly, hypoxia induces the activation of Src/PI3K proteins, stabilizing HIF-1α expression levels while it phosphorylates and activates STAT3 [137]. HIF-1α and STAT3 bind to VEGF promoter, forming a complex with CBP/p300 and Ref-1/APE, thus having a synergic effect on the overexpression of VEGF, which is essential to tumoral cell survival in hypoxia and inflammation [132,137]. Additionally, downstream, the expression of Akt as a consequence of STAT3 activation leads to upregulation of HIF when the pathway is stimulated by growth signals such as IL-6, contributing to the upregulation of VEGF [133].

Furthermore, hypoxia seems to induce the activation of other members of the STAT family, such as STAT5 [138,139]. Hypoxia induces the phosphorylation of STAT5, leading to an increment in its binding to DNA, more specifically, in the gene promoter; in this manner, hypoxia would induce the expression of proliferation genes such as cyclin D1 [140].

**Figure 5:**
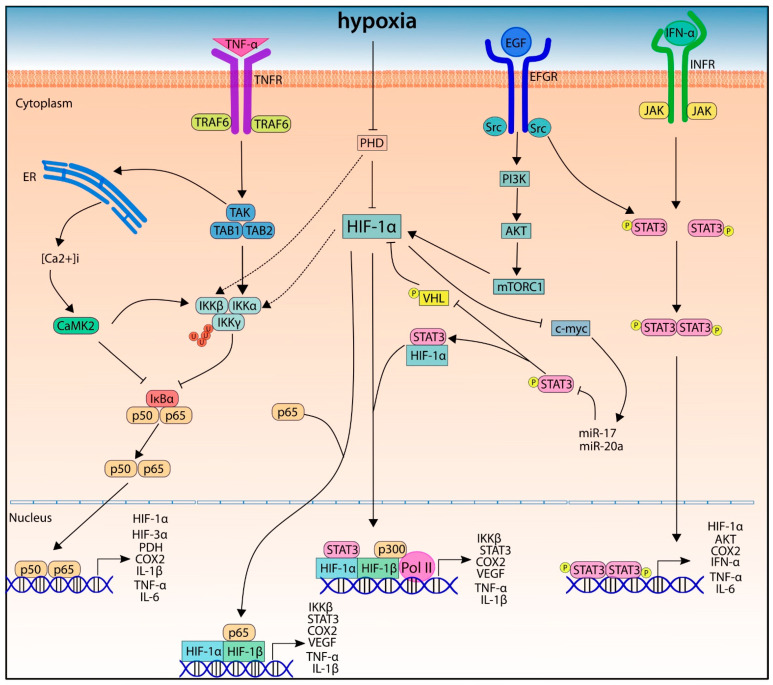
Intercommunication between HIF, NF-κB, and STAT pathways during hypoxia. Hypoxia induces NF-κB activity through CaMK2 and the presence of TAK and IKK, leading to the sumoylation of IκBα. TNF-α also increases HIF-1α levels through the NF-κB pathway. Hypoxia through HIF-1α also induces phosphorylation of the inhibitory IκBα for its degradation and thus activation of NF-κB. Moreover, hypoxia inactivates the PHD hydroxylation over IKKβ, which conducts IKKβ to degradation; instead, IKKβ phosphorylates IκBα to finally activate NF-κB. STAT3 induces HIF-1α expression and avoids its degradation, even independently of hypoxia, inducing gene transcription. STAT3 and HIF-1α interact and recruit coactivators to induce gene transcription, including VEGF. Hypoxia induces the expression of Src, which leads to STAT3 activation and, in consequence, HIF-1α stabilization. Akt is also activated by STAT3, and as feedback, induces HIF-1α expression. miR17 and miR20a inhibit differentiation and STAT3 activation. However, HIF-1α inhibits these miRNAs and avoids their effects (see more details in the main text).

The intercommunication between STAT and NF-κB pathways with hypoxia reflects the complexity of the hypoxic effects in cancer (Figure 5). It has been proposed that both STAT3 and NF-κB promote tumoral initiation, promotion, and progression through overexpression of pro-tumorigenic genes such as TNF-α, fibroblast growth factor (FGF) 2, VEGF, IL-1β,-8,-11,-12,-17,-22,-23, IFN-δ, vascular cell adhesion-1 (VCAM-1), ICAM-1, C-X-C Motif Chemokine Receptor (CXCR) 4, M-CSF, MCP-1, metalloproteases (MMP) 9,-2, Cyclin D, and c-FLIP [141,142,143,144]. Some target genes of NF-κB, like IL-6, are activators of STAT3, which acetylates RelA, retains NF-κB in the nucleus, and thus prolongs its activity in cancer cells [145]. Ivanova and Perkins have reported that hypoxia induced the translocation of RelA and IκBα from the cytoplasm to the mitochondria. Interestingly, the ROS produced during hypoxia is linked to the recruitment of RelA and IκBα to mitochondria and the authors suggested that STAT3 could facilitate this process [146]. In fact, mitochondrial RelA would regulate mitochondrial energy production and oxygen consumption [147].

The biological processes involved in the intercommunication of hypoxia and STATs are complex. Recently, hypoxia has been related to the induction of pyroptosis through the participation of STAT3 [148]. Pyroptosis is another type of programmed cell death characterized by the participation of gasdermins, a group of proteins that induce the formation of pores in the membrane and the release of pro-inflammatory molecules. Gasdermin D (GSDMD) is activated after it is cleaved by caspases 1, 4, 5, and 11, a group of caspases that do not participate in apoptosis [149]. Interestingly, PD-L1, usually known as a suppressor of anti-tumoral immunity, translocates into the nucleus in hypoxic conditions; however, this is not dependent on HIF-1α. Moreover, this effect was mediated by the phosphorylation in Y705 of STAT3, which interacted with PD-L1. Both nuclear PD-L1 and Y705-STAT3 induce the expression of gasdermin C (GSDMC), which will be cleaved by caspase-8. The stimulation with TNF-α would then induce pyroptosis instead of apoptosis in hypoxia, explaining the mechanism of how TNF-α induces tumor necrosis in hypoxia (Supplementary Figure S1) [148].

Another mechanism underlying the communication between hypoxia and inflammation could be mediated by microRNAs (miRNAs). miRNAs are non-coding RNAs that are 21–23 nt long and that regulate the gene expression at a post-transcriptional level and play an important role in the regulation of inflammation in cancer [150]. In fact, it has been reported that miRNAs regulate the expression of STAT3 through transcription factors such as NF-κB or HIF-1α, with the latter closely related to hypoxia in cancers such as leukemia or colon cancer [151,152]. Interestingly, it has been documented that the induction of hypoxia in acute myeloid leukemia (AML) and the subsequent expression of HIF-1α leads to cell differentiation [153]. In this sense, it has been reported that HIF-1α, through the downregulation of c-Myc, represses miR17 and miR20a, which in turn decreases the expression of STAT3 and p21 in a model of AML [151]. In these cells, the hypoxic microenvironment represents, in the case of HIF-1α activation, a protective system that could be a potential treatment strategy. In the case of colon cancer, the knockout of miR-139-5p activates STAT3, facilitating a pro-tumoral state [150,152].

## 3. Cellular Mediators of Inflammation Modulated by the Hypoxic Response as Inductors of Cancer Progression

**Figure 6:**
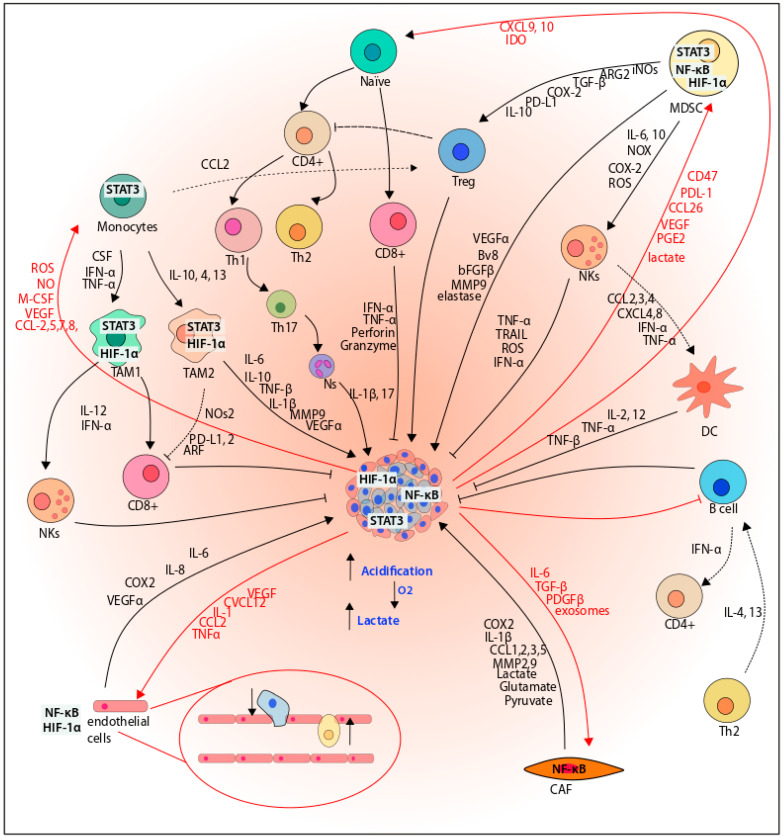
Intercommunication between tumoral and immune cells in hypoxia. Hypoxia triggers the activation of HIF-1α, NFκB, and STAT pathways, boosting the transcription and releasing of chemotactic factors (CXCL12, VEGF, and CCL-2, -5, -7, -8, -12 and -26), pro-inflammatory cytokines (TNF-α, TNFβ, and IL-1,-6) as well as the secretion of exosomes, PD-L1, lactate, PGE2, ROS, and NO, among other molecules that targets immune cells. Red arrows represent the stimulation of immune cells, whereas red truncated arrows represent inhibition. These signal molecules activate several mechanisms that result in the infiltration of pro-tumor immune cells, such as TAMs, MDSCs, CAFs, Ns, and Treg cells, to the tumor, which suppress the anti-tumor response of CD8+ cells, B cells, NK cells, and dentric cells (DCs). Immune cells, in response, release IL-1β, -4, -6, -8, -10, -12, -13, -17, CCL-1, -2, -3, -5, -22, TNFα, TNF-β, IFN-γ,M- CSF, VEGF, bFGFβ, PDGF, PDL1, MMP-2,-9, Arg-1, NOX2, and COX-2 as well as the release of glutamate, pyruvate, and lactate among other molecules, resulting in the promotion of inflammation, metabolic adaptations, growth of tumors, epithelial to mesenchymal transition, angiogenesis, migration, invasion, metastasis, and resistance to chemo, radio, and immune therapy. Black arrows represent the stimulation of tumoral cells, whereas black truncated arrows represent inhibition. See main text for more details.

Hypoxia is not restricted to only tumoral cells; it is part of all TMEs, including the cellular mediators of inflammation. We explore in detail the effects of hypoxia in each inflammatory cellular component and how it participates in cancer development (Table 1, Figure 6).

### 3.1. Macrophages

Immune cells as macrophages and neutrophils are attracted to zones with low levels of oxygen, such as wounds, but also to hypoxic tumoral regions. The presence of macrophages is a well-characterized phenomenon that is associated with cancer, and there has been much study of the presence of the macrophages and the correlation with a bad prognosis [211].

Monocytes are recruited to the tumor by chemokines such as CCL2, CCL5, CCL7, CCL8, CXCL12, VEGF, and M-CSF, and become TAMs [154,212]. TAMs release cytokines such as IL-8 or TGF-β and pro-angiogenic factors such as VEGF and FGF2, which induce revascularization to bring nutrients to the zones without irrigation, allowing tumoral progression. In addition, the intercommunication between tumoral cells promotes the release of endothelin-1 and endothelin-2 and induces the secretion of MMP2, MMP7, or MMP9, increasing the metastasis. In addition, TAMs generate an immuno-protective environment, inhibiting the activation of T cells [158].

In addition to the presence of macrophages in the tumor, the subtype of the macrophage is also crucial. Macrophages have a specific subtype, named M1 or M2. The M1 phenotype is activated in the presence of microorganisms or IFN-γ and is characterized by high secretion of IL12/IL-23 but low IL-10. M1 cells have an inflammatory profile and eliminate pathogens and debris and therefore are considered as a tumor suppressor phenotype. In this sense, the M1 phenotype releases IL-12, IFN-γ, TNF-α, IL-1β, ROS, and nitric acid, hence promoting the recruitment and activity of NK cells and CD8 + T cells in the tumor microenvironment to induce an anti-tumor immune function and inflammation. Furthermore, IL-1β, TNF-α, and IL-1 activate NF-κB through binding to their corresponding receptors [213].

On the contrary, the M2 phenotype is related to an anti-inflammatory function, linked to the release of proliferative signals and the revascularization of the tumor. Importantly, the tumoral cells release cytokines that induce the polarization of the TAMs to the M2 phenotype [154,214]. In this sense, the M2 phenotype is induced by signals such as IL4, IL10, IL-13, TGF, M-CSF, and glucocorticoids, among other cytokines. Activated M2 macrophages produce high levels of IL-10, IL-6, and EGF, which activate the JAK/STAT3 signaling. Moreover, M2 macrophages release CCL22, but in contrast to the M1 phenotype, produce low levels of IL-12 [213,215]. CCL22 promotes the infiltration of Treg cells to the tumor, releasing IL-10, which suppresses the antigen presentation function of dendritic cells and the anti-cancer response of CD8+ T cells [213]. On the other hand, studies have reported the overexpression of PD-L1 in M2 macrophages, which binds to CD8+ T cells through the programmed death-1 (PD-1) receptor, thereby achieving the tumor immune escape [213].

However, it is worth noting that there is a combinatorial spectrum of macrophage populations, other particular populations, such as CD169+ macrophages and TCR+ macrophages, have been recognized. Moreover, some groups reassign tumor-associated macrophages as a particular population with a potential switch from the M1 to M2 profile and vice versa, which can be used as a therapeutic approach [216].

In this context, the hypoxic microenvironment induces the M2 phenotype, promoting the survival of tumoral cells. In addition, the low concentration of oxygen modulates the expression of hypoxia-sensitive genes in M2 TAMs, particularly those related to angiogenesis [159]. For example, M2 TAMs secrete IL-6 with the corresponding expression of the IL-6 receptor in the tumoral cells, activating the STAT3 pathway to promote survival in the tumoral cells located in the hypoxic region [162]. Moreover, in a hepatocarcinoma model, hypoxia, as well as the necrotic debris of tumoral cells, induced IL-1β release from M2 TAMs, which up-regulated the expression of HIF-1α through the NF-κB/COX-2 pathway. Interestingly, this profile induces a mesenchymal phenotype in the tumoral cells, characterized by an increment of vimentin and a reduction of E-cadherin [66]. However, even the induction of M2 TAM phenotype by hypoxia is clear; in some cases a pro-inflammatory profile has also been reported [66,163,217].

Importantly, HIF is a key regulator in the bidirectional response between the macrophages and the tumoral cell; therefore, the presence of macrophages in hypoxic conditions has been linked to a bad prognosis. The hypoxic microenvironment, through HIF as the principal mediator, induces the secretion of chemokines, which recruit macrophages and restrain them in the tumor. For example, HIF induces the expression of CCL2 in pancreatic ductal adenocarcinoma cells, which induces the recruitment of macrophages to the tumor [155]. Other chemokines related to monocyte recruitment in hypoxia are CCL5, VEGF, EMAP II, and endothelin-1 and 2 [156]. It has been reported that hypoxia, through HIF-1α, induces the up-regulation of CXCR4 in TAMs but also in endothelial and cancer cells; this axis regulates the migration of the different cells that integrate the TME [157]. In addition, hypoxic macrophages show elevated secretion of CCL4 and promote MMP9 expression in glioblastoma cells, while the hypoxia up-regulates the CCR5 expression [161]. Even more, there is also a clear effect between the recruitment and activation of the tumoral macrophages and the presence of HIF. It seems that HIF benefits the recruitment and the M2 phenotype polarization. For example, the induction of HIF-1α leads to IL-10 expression and, as a consequence, the polarization of M2 macrophages in an inflammatory environment due to obesity [164]. Furthermore, it seems that hypoxia through HIF is a major regulator of the M2 polarization in macrophages, although in some cases it seems that HIF could support the M1 phenotype and also be protective [167].

Recently, it has been reported that hypoxia can contribute to immune evasion avoiding macrophage phagocytosis through the regulation of CD47 expression in cancer cells. CD47 is a cell-surface protein that binds to the signal regulatory protein α (SIRPα) expressed in macrophages, preventing phagocytosis. Moreover, HIF-1α activates the transcription of CD47 in breast cancer cells in hypoxia [218]. As a consequence, anti-CD47 antibodies have been proposed as therapeutic alternatives, with favorable results [219].

Macrophages can also recognize and eliminate tumoral targeted cells marked with antibodies, in a mechanism known as antibody-dependent cellular phagocytosis (ADCP). Fcγ receptors expressed in macrophages recognize and bind to the Fc regions of antibodies, activating a signaling cascade, resulting in phagocytosis being proposed as a possible strategy for immunotherapies [220]. However, ADCP is impaired by the inhibitory receptor FcγRIIb. Interestingly, HIF-1α induced the transcription of FcγRIIb, contributing to the resistance to immunotherapy in hypoxic tumors [221].

Delprat and colleagues make a distinction and propose that intermittent hypoxia, also known as cycling hypoxia (cyH), exacerbates the inflammatory response in the TME. In particular, cyH induces a pro-inflammatory phenotype in unpolarized M0 and amplifies this profile in M1 macrophages through the activation of the c-jun/p65 signaling pathway [222].

Even though the principal and best-characterized mediator of hypoxia is HIF-1α, some evidence suggests that HIF-2α also has an important role in the modulation of the macrophage function. Mice with myeloid cells lacking HIF-2α expression show a diminution in the infiltration of TAMs, apparently through the modulation of the expression of M-CSF receptor and CXCR4, hence reducing the tumoral progression [223]. It seems that the different isoforms of HIF could be induced from a different stimulus. For example, Th1 cytokines benefit the M1 phenotype through HIF-1α, whereas Th2 induces the M2 phenotype through HIF-2α and regulates the NO production [224].

Moreover, the activating transcription factor (ATF4) is stimulated by stress signals, including hypoxia [225]. In this sense, macrophages in hypoxic conditions express not only HIF-1α and HIF-2α, but also ATF4 [226,227]; more importantly, ATF4 is capable of inducing the recruitment of M2 macrophages to the tumor in a hemangioma model [228].

Tumoral hypoxia produces metabolic modifications, including the activation of the glycolytic pathway, the inhibition of oxidative phosphorylation (OXPHOS) in mitochondria, and the accumulation of lactate. HIF-1α is a major inductor of glycolysis, which allows M1 macrophage differentiation. Furthermore, OXPHOS and glycolysis are required for M2 macrophage differentiation. The regulation of this switch would participate in the anti-tumoral protection [165]. On the other hand, lactosis in solid tumors is related to the differentiation of monocytes to macrophages. Moreover, TAMs exhibit an inflammatory profile, such as CXCL1, CCL18, and CCL24, which favor the accumulation of immunosuppressive myeloid cells, T cells, and monocytes, as well as M-CSF, which induces monocyte recruitment and M2 TAM differentiation, conferring a protumor and inflammatory M2 phenotype [163].

The effects of angiogenesis induced by the TAMs go beyond the release of angiogenic factors. Recently, it has been reported that macrophages themselves could form non-endothelial vascularity around the tumor, derived from the hypoxic stimulus [160]. This flexibility is evidence of the multiplicity of the tumoral environment to facilitate tumoral progression.

The regulation of the TME is related also to different levels, including miRNAs. These non-coding RNAs regulate different functions in the cell, and it seems that they are involved in the regulation of the immune cells that surround the TME; miRNAs have been extensively reviewed elsewhere [150]. A recent example is miR-155 and miR-21, which regulate the TAM function and reduce tumoral growth [229]. Interestingly, miR-17 and miR-20a regulate the expression of HIF2-α, even in normoxic conditions, and induce a pro-angiogenic response in TAMs [230]. In another example, hypoxia induces the expression and secretion of miR-940 in exosomes of epithelial ovarian cancer, which are delivered in macrophages and lead to M2 polarization and thus cancer progression [166].

### 3.2. Fibroblasts

The fibroblasts are the most abundant cell type in the stroma and usually secrete extracellular matrix in wounds to achieve reparation, following the evolution in the inflammation process. However, in the complexity of the TME, the CAFs acquire a pro-tumoral function, including the induction of metastasis through the remodeling of the extracellular matrix (ECM) and the secretion of angiogenic and proliferative factors [231]. In this sense, tumor cells induce the transformation of stromal fibroblasts into pro-tumorigenic CAFs through the release of IL-6, TGFβ, PDGF, exosomes, and specific TME stimulus, including hypoxia, acidification, and oxidative stress, which promote the recruitment and activation of fibroblasts [168,169,232].

It has been demonstrated that tumoral cells induce the transformation of fibroblasts isolated from an initial hyperplastic state into CAFs with pro-inflammatory properties. CAFs, through the IL-1β/NF-κB signaling axis, induce the transcription of COX-2, IL-1β, IL-6, CCL3, CXCL1, CXCL2, CXCL5, MMP3, and MMP12, leading to macrophage recruitment, angiogenesis, tumoral growth, and metastasis [233]. In addition, the treatment of fibroblasts with pro-inflammatory leukemia inhibitory factor (LIF) induces an epigenetic switch, through p300 histone acetyltransferases that acetylate STAT3. Then, STAT3 induces the transcription and activation of DNMT3b methyltransferase, which inhibits the tyrosine phosphatase (SHP-1) expression, resulting in constitutive activation of the JAK1/STAT3 pathway and leading to extracellular matrix remodeling and collective migration and invasion of neoplastic cells [234]. Moreover, CAFs impair tumor immunity due to the induction of massive infiltration of myeloid cells into the tumor stroma. CAFs also decrease the proliferation and activation of T cell cytotoxicity [235,236].

However, as previously mentioned, hypoxia and oxidative stress induce the differentiation of CAFs and lead to metastasis. In this regard, ROS induces the conversion of stromal fibroblasts into migrating myofibroblasts, due to the accumulation of HIF-1α, which promotes the activation of the CXCL12/CXCR4/Rho A signaling pathway. These characteristics were found in HER2-human breast carcinomas, which present high rates of cell proliferation, neovascularization, and metastasis [170].

Another approach showed that breast cancer cells lead to the transformation of fibroblasts to CAFs through an autophagic mechanism. Tumoral hypoxia activates HIF-1α and NF-κB transcription factors, which drive the autophagic degradation and loss of Cav-1 with the consequent stabilization of CAFs, thus exerting pro-tumoral actions that will benefit the metastasis of the cancerous cells [171]. In this regard, CAFs play an important role in the regulation of cancer metabolism, primarily through the secretion of metabolites and the generation of a stiffer and fibrotic ECM, which in turn affects cancer cell metabolism. In this sense, it has been proposed that the activation of catabolic and autophagic pathways in CAFs increase the production of metabolites such as pyruvate, lactate, glutamate, and ketone bodies that are available to the surrounding cancer cells; moreover, this correlates with a higher invasive and resistance capacity of these cells through a reciprocal metabolic reprogramming [235,237].

However, the effects of hypoxia on CAFs could seem contradictory. For example, the hypoxic conditions seem to reverse the pro-tumoral phenotype of the CAFs, observed with the impairment of the ECM remodeling and CAF-induced cell invasion, through a mechanism dependent on the inhibition of PHD2 through low oxygen levels and the consequent stabilization of HIF-1α in a breast cancer model, making PHD2 in CAFs a probable therapeutic target [172,173].

### 3.3. Natural Killer Cells

NK cells are lymphocytes from the innate response associated to antitumor protection [238,239,240]. In this sense, activated NK cells recognize and eliminate tumoral cells without the requirement of previous recognition. For instance, NK cells recruit type 1 dendritic cells to the tumor and promote T1 cell polarization through the release of CCL2, CCL3, CCL4, CCL5, XCL1, and CXCL8, thus inducing anti-tumoral protection [241,242].

NK cells also release granules with cytotoxic content, such as IFN, TRAIL, and FasL. NK cell-through killing is executed by FASL and TRAIL on the NK cell surface, which leads to apoptotic cell death in target cells. Furthermore, the NK cells contain lytic granules with perforin, a protein that causes membrane pores, and granzymes, a family of serine proteases, which are exocytosed to lysed target cells [243]. These properties seem very beneficial to developing new cancer immunotherapies [239].

Cellular acidity derived from anaerobic metabolism due to hypoxia has detrimental effects on NK and T cells [174]. In this sense, the TME facilitates the tumor evasion of the immune system. As the tumor grows and hypoxia is established, cancer cells change their metabolism to a glycolytic profile; in this context, cells metabolize pyruvate to lactate through LDH, inducing an acidic environment. The high levels of lactate and pyruvate induce the accumulation of HIF-1α, even in the presence of oxygen, and as a consequence induce the expression of genes from the HIF-1α pathway, including those that lead to a glycolytic environment [244]. Moreover, LDHA, a target of HIF-1α [245], leads to lactate accumulation, which in turn impairs the function of NK cells and T lymphocytes [175]. High levels of lactate inhibit the production of IFN-γ, apparently through the inactivation of the nuclear factor of activated T cells (NFAT). A diminution in the viability of both NK cells and T lymphocytes was detected in this lactic environment [176]. Moreover, evidence shows that the accumulation of lactate and this acidic environment impairs the cytotoxicity action of NK cells, which is favorable to tumoral cell proliferation and protection from the immune system [175,177].

As previously discussed, hypoxia has a crosslink with the STAT pathway. It has been reported that hypoxia induces impairment of the NK cell cytotoxicity against tumor cells, and this effect is associated with the reduction in the phosphorylation of ERK and STAT3. The ERK and STA3 phosphorylation depend on the activation of the tyrosine phosphatase Src homology region 2 domain-containing phosphatase-1 (SHP-1) by hypoxia; in fact, the pharmacological inhibition of SHP-1 by TPI-1 allows a partial recuperation of NK cytototoxicity [246].

NK cells express the NKG2D transmembrane receptor that binds to their ligand (NKG2DL), present in the tumor cells; thus, NK cells can recognize and kill the tumoral cells [247]. However, the microenvironment may modify the expression of NKG2D receptor or ligand, avoiding tumoral immunosurveillance. For example, in a model of resistant prostate cancer, hypoxia decreased the expression of UL16 binding protein, which is a member of the NKG2D family, and MHC class I chain-related proteins A and B (MICA/MICB). Apparently, hypoxia also induced the expression of PD-L1, which could be blocked with inhibitors of the JAK/STAT3 axis to re-activate the cytotoxic action of NK cells [178]. In accordance with this, another group reported a decrease in MICA expression in osteosarcoma cells, inhibiting the NK cell antitumoral cytotoxicity [181].

In a model of pancreatic cancer, Ou and colleagues found that hypoxia leads to a significant presence of soluble major histocompatibility complex class I, chain-related (sMICA), related to the shedding of membrane MIC (mMICA) from the tumor cell membrane to a soluble form. In addition, the expression of NKG2D was downregulated, avoiding the NK cytotoxicity over tumoral cells [179,180]. Alternatively, the shedding of sMICA could be inhibited by nitric oxide signaling, counteracting the hypoxic effect [248].

Moreover, the participation of dysregulated circRNAs and miRNAs such as circ_0000977/miR-153 was linked to the regulation of HIF-1α. In hypoxia, an increase in the presence of circ_0000977 was detected and correlated with an upregulation of HIF-1α, while miR-153 had the opposite action. More interesting, miR-153 was able to bind to circ_0000977 and HIF-1α, establishing a possible mechanism of regulation. The authors proposed that hypoxia leads to an increase of circ_0000977, which inhibits miR153 and releases its repression over HIF-1α and A Disintegrin and Metalloproteinase Domain 10 (ADAM10). Then, a transition from membranal MICA to soluble MICA occurs; sMICA then bounds to NKG2D over NK cells, decreasing their stimulation and leading to the immune escape of the tumoral cells [180]. In contrast, Sarkar and colleagues suggested that the detrimental action of hypoxia over NK cells could be counteracted with a pre-activation of NK cells by IL-2 using a model of melanoma. Apparently, in this model, they did not find alterations in the levels of MICA/B, HLA-ABC, and ULP1-2 under hypoxia [249].

Hypoxia decreases the levels of other NK cell membrane receptors, such as NKp46, NKp30, and NKG2D, as previously mentioned. Then, NK cell ability to eliminate tumor cells would be reduced [250]. Interestingly, CD16+ NK cells recognize the Fc of immunoglobulins attached to target cells, establishing a mechanism known as antibody-dependent cellular cytotoxicity (ADCC) to eliminate them. Moreover, hypoxia seems not to particularly affect the ADCC mechanism. Solocinski and colleagues used a model with NK cells that overexpress CD16 receptor and showed that these cells maintain their cytotoxic capacities in hypoxic conditions, being a potential strategy for immunotherapy in cancer [251]. It has been observed that hypoxia did not decrease the expression of CD16 receptor in NK cells, contrary to other activating receptors [250].

Metabolic stress such as in the hypoxic microenvironment is also related to the release of eADO. Hypoxia induces dephosphorylation of adenosine triphosphate (ATP) through nucleoside triphosphate dephosphorylases (NTPD) and upregulates the activity of ectonucleotidases such as CD39 and CD73, increasing the adenosine levels. Then, adenosine modulates immune cells through the activation of adenosine receptors such as A1, A2A, A2B, and A3, located in the immune cells [252]. Particularly in NK cells, adenosine seems to inhibit granule exocytosis and the lytic activity of NK cells. An extended review of this topic has been published recently [84].

The TME depends on the intercommunication between the tumoral cells and surrounding cells, and hypoxic signals model this communication in both directions. In a revealing work, Krzywinska and colleagues demonstrated that the deletion of HIF-1α in NK cells impairs their cytotoxicity but decreases tumoral growth [182]. Moreover, the NK cells with HIF-1α deletion were associated with a deficient vasculature in the tumors and consequently with metastasis. The absence of HIF-1α in NK cells was associated with a decreased expression of the soluble form of VEGF receptor 1 (sVEGFR1) in the tumors from mice with NK cells with HIF-1α deletion. Interestingly, sVEGFR1 sequestrates VEGF and modulates its bioavailability. In this case, the secretion of HIF-1α from NK cells leads to the expression of sVEGFR1 and avoids aberrant angiogenesis in tumors through the regulation of bioavailability of VEGF [182].

### 3.4. Myeloid-Derived Suppressor Cells

The MDSCs are immature myeloid cells that originated in bone marrow from immature myeloid cells (IMCs) and differentiate in dendritic cells, macrophages, and granulocytes. MDSCs are characterized by their suppressive activity over T cells; moreover, MDSCs have been associated with the inhibition of antitumoral immunity and the promotion of tumor progression [190,253,254]. This response is related to the release of pro-angiogenic molecules, including VEGF-A, Bv8, bFGF, elastase, and MMP9 [255,256].

The MDSC are classified according to their lineage into two groups: monocytic MDSCs (M-MDSCs) and polymorphonuclear MDSCs (PMN-MDSCs). MDSCs are activated by several stimuli, such as tissular damage, pathogens, chronic infections, autoimmune diseases, and inflammatory signals in the context of cancer [254]. MDSCs are present in the majority of cancer types, promoting the progression of the tumor and inhibiting the antitumoral immunity mediated by T cells; in fact, this represents an obstacle to the immunotherapies against cancer [190,257].

MDSCs produce high levels of ROS and nitric oxide, which hinder the infiltration, activation, and apoptosis of T cells [255,258,259]. MDSCs also produce ROS and peroxynitrite, which induce the nitration of CTR/CD8 receptors, reducing their interaction to cognate antigen-MHC complexes and thus inhibiting CD8+ T cells [258].

Tumors release prostaglandin E2 (PGE2), inducing the nuclear accumulation of p50 NF-κB in M-MDSCs. Furthermore, p50 facilitates the binding of STAT1 to DNA to activate the transcription of nitric oxide synthase (NOS2) and other genes dependent on IFNγ activation, promoting an immunosuppressive phenotype of MDSCs [260]. Furthermore, MDSCs induce the expression of immunosuppressive cytokines and mediators, such as COX2, arginase (Arg) 2, inducible nitric oxide synthase 2 (iNOS2), PD-L1, TGFβ, IL-10, and CCR5, leading to activation and infiltration of Tregs as well as NK cell inhibition [255,259].

In this context, intratumoral hypoxia induces the expression of immunosuppressor molecules such as CD47 and PD-L, cytokines such as CCL26, and proteins such as ectonucleoside triphosphate diphosphohydrolase 2 (ENTP2/CD39L1), which depend on HIF activity. These molecules act as chemoattractants to recruit and increase the MDSCs in the tumor and to inhibit indirectly the cytotoxicity produced by the NK cells, prevent immune surveillance, and increase tumoral growth and progression [183,184,185]. Moreover, the overexpression of ENTPD2 leads to an increase of AMP, which prevents the differentiation of MDSC and facilitates its accumulation in the tumor [185]. In this sense, some growth factors such as VEGF also act as a chemoattractant of MDSC. MDSC secretes VEGF, inducing angiogenesis [184,190,191,192].

The overexpression of CD45 protein tyrosine phosphatase (PTP) in MDSCs exposed to hypoxia in the tumor site promotes the inactivation of STAT3, resulting in the M-MDSC differentiation to TAM [188]. Moreover, hypoxia induces a shift from the dimeric to the monomeric form of CD45 phosphatase, the more active form of this protein. Apparently, when M-MDSCs migrate to the tumor, the hypoxia induces the overexpression of sialin, a sialic acid transporter, facilitating the transport of sialic acid to the membrane, allowing its binding to CD45 phosphatase and preventing its dimerization. The activation of CD45 phosphatase then leads to inactivation of STAT3, thus facilitating MDSC differentiation into TAM [188].

Likewise, it has been described that HIF-1α mediates the differentiation of MDSCs to TAMs through the upregulation of iNOS and Arg1. Consequently, the expression of nicotinamide adenine dinucleotide phosphate oxidase (NOX) 2 and ROS in MDSCs decreases, leading to the suppression of antigen-specific and nonspecific T cell activity [189].

In addition, hypoxia can increase the MDSC activities through HIF1α/miR-210 signaling. miR-210 enhances the Arg1 activity and NO levels, without alteration of ROS, IL6, IL10, and PD-L1 levels [261].

It has been observed that lactate localized in hypoxic areas induces the recruitment of MDSCs and inactivates NK cells in the tumor site, resulting in a suppression of the anti-tumoral response [175]. In fact, it has been demonstrated that lactate derived from tumor cells upregulates the expression of PD-L1 [262]. In this sense, an essential mechanism of immunosuppression is mediated by HIF-1α/PD-L1 signaling in TAM, MDSCs, and dendritic cells by hypoxia [186,187].

In addition, it has been reported that the upregulation of PD-L1 transcript in hypoxia is mediated by the cooperative interaction of PKM2/HIF-1/p300 on the PD-L1 promoter [187]. Furthermore, PD-L1 on cancer cells induces the glycolytic process through Akt/mTOR signaling, inducing an immunosuppressive tumor microenvironment [263]. The blockade of PD-L1 with a monoclonal antibody in hypoxia reverts the immunosuppressive response, increasing the proliferation and activity of T cells accompanied by the downregulation of IL-6 and IL-10 in MDSCs. Thus, the inhibition of PD-L1 in tumoral hypoxia is proposed as a potential treatment [186].

### 3.5. T Cells

T cells are also important for adaptative immunity and usually mature in the thymus. Naïve T cells are activated after the interaction of the antigen-T cell receptor to differentiate in CD4+ or CD8+. In the case of CD4+ T cells, they can be divided into helper (Th) and regulatory T cells (Treg). Th cells, in turn, are subdivided into distinct subtypes and activate different cellular types: Th1 releases IFN-γ and IL-2 to activate macrophages and cytotoxic T cells; Th2 secretes IL-4 and IL-13 and activates B cells, while Th17 releases IL-17A to recruit neutrophils and macrophages. Treg suppresses the immune response. CD8+ T cells or cytotoxic T cells release pro-inflammatory cytokines such as INF-γ and TNF-α as well as cytotoxic molecules such as perforin and granzymes and can eliminate cells that are infected.

After antigen stimulus, T cells proliferate using aerobic glycolysis, as c-Myc and HIF are important regulators to control glycolysis and glutaminolysis [264]. In particular, hypoxia can also affect the functions of T cells, apparently supporting the antitumoral response. Palazon and colleagues demonstrated that HIF-1α induces a glycolytic profile and increases the migration of CD8+ T cells. Moreover, an increase in the cytotoxic activity of CD8+ T cells was observed, linked to the increase of costimulatory molecules such as CD137, OX40, GITR, PD-1, TIM3, and LAG3 and the production of granzyme B. VEGF-A expression, a target gene of the HIF pathway, is also correlated with tumoral vascularization [193]. Other groups have reported that hypoxia enhanced the lytic activity and function of cytotoxic T lymphocytes (CTLs), related to the increase of granzyme-B [194,195,196]. On the contrary, another group reported that HIF2α and not HIF1α induces cytotoxic differentiation and cytolytic activity over CD8+T using retroviral vectors for ectopic expression of HIF1α and HIF2α in CD8+ T cells [197].

However, there is controversy around hypoxia and its adverse effects in cancer. For example, hypoxia has been linked to radiotherapy resistance. In this sense, hypoxia downregulates the MHC I expression of tumor cells, avoiding their recognition by CD8+ T cells. In addition, hypoxia seems to reduce the levels of CXCL9, CXCL10, and IDO, whose expression is stimulated by IFN-γ, as a potent cytokine with antiproliferative actions [198]. Thus, hypoxia reduces the proliferation and antitumor functions of CD8+ T cells, preventing the antitumoral immune response [199].

Interestingly, the chronic response of T cells to a prolonged stimulus such as cancer has been defined as T cell exhaustion [265]; moreover, the density of exhausted T and B cells, as well as T-cell exhaustion–related genes like PDL1, B7H3, FOXO1, and PRDM1, correlates with a high expression of HIF-1α in glioblastoma [200]. HIF-1α and VEGFA promote the differentiation of the CD8+T cells to exhausted T cells, highlighting their proangiogenic profile [201].

Recently, a subset of T cells that do not recirculate, known as tissue-resident memory T cells (TRM), was identified. TRM cells express CD69 and CD103 in some tissues and secrete different cytokines according to their residency as TGF-β, IL-15, Type I IFN, and IL-12, contributing antitumoral activity and better prognosis. In this sense, the presence of hypoxia and TGF-β1 induce the differentiation of CD8+ T cells to TRM [202]. Moreover, hypoxia promotes the antitumor effect of TRM cells, as was evaluated using a VHL-deficient CD8+ T cell tumoral model [266]. It has been reported that a synergism exists between the expression of TGF-β and hypoxia to induce the differentiation of CD8+ T cells in the TRM, which contributes to antitumoral response [267].

Moreover, another subset of T cells known as gamma delta (γδ) T cells, found in peripheral blood, has been described as expressing characteristics of innate and adaptive immune responses and has been postulated as a candidate for immunotherapy in cancer [268]. Interestingly, hypoxia drives a reduction in γδ T cell cytotoxicity against oral tumoral cells, due to a decrease in the degranulation of the cytotoxic contents and inducing their differentiation to γδT17, which releases IL-17, a pro-tumorigenic cytokine [203].

A precondition of anti-angiogenesis therapy in cancer using anti-VEGF antibodies demonstrated improvement of the function of CD8+ T cells, apparently linked to an increase in hypoxia due to the inhibition of VEGFR2 signaling [269]. In another immunotherapy model, CTLs were preincubated in 1% oxygen and showed that the package of granzyme-B per granule was more efficient and thus their cytotoxic effect improved. This correlates with a better regression of the tumor in vivo in a model of melanoma [270].

Besides the HIF-1α pathway, other signaling pathways can participate and modulate T cell function. For instance, delta-like 1 (DLL1), is a ligand of the Notch pathway that is expressed in endothelial cells and has been linked to aberrant vascularization in cancer, acting as a compensator of tumoral hypoxia. When DLL1 is overexpressed in breast and lung cancer lines, it induces a normal vascularization around the tumor and the activation of CD8+ T cells, which could be useful in cancer immunotherapy, ameliorating the distribution of the antitumoral drugs [271].

Moreover, it has been demonstrated that HIF-1α binds to the FoxP3 promoter and induces its expression. FoxP3 is a crucial regulator of T cell differentiation into Treg, which deploys anti-inflammatory mechanisms associated with a bad prognosis in cancer [204,205]. Recently, the role of HIF-2α in the stabilization of Treg has been characterized. In fact, the knockout of HIF-2α in Treg results in an inability to suppress inflammation; moreover, the implantation of Tregs with the knockout of HIF-2α resulted in a restriction of the tumoral growth in an in vivo model of colon carcinoma [272].

### 3.6. B Cells

B cells are responsible for the humoral immune response. Briefly, B cells mature in bone marrow, and then they exit to the bloodstream and ganglia, where are exposed and recognize antigens; later, they differentiate into plasmatic or memory cells, both with the capacity to produce antibodies. Normally, B cell development occurs in the bone marrow and implies their maturation and selection through the B cell receptor (BCR). Interestingly, the regulation of HIF-1α is essential for the normal maturation of B cells. A sustained expression of HIF-1α leads to developmental arrest and BCR defects due to suppression of the proapoptotic BCL-2-interacting mediator of cell death (BIM) [273]. Additionally, HIF-1α induces a glycolytic profile in immature B cells [274,275].

Several studies have reported the capacity of B cells to promote an antitumoral function [276,277]. For instance, B cells enhance T cell antitumoral response by acting as antigen presentation cell (APC), or they release effect cytokines as IFN-γ to polarize T cells towards a Th1 or Th2 phenotype [278]. It has been reported that both CD20+ B cells and CD8+ T cells cooperate to induce antitumor immunity in ovarian cancer, increasing patient survival significantly [276]. In another example of anti-tumoral response, margin infiltrating B cells mediated direct cytotoxicity through the secretion of IFN-γ, TRAIL, and granzyme B on hepatoma cells [279]. Furthermore, human B cells stimulated with CpG-oligodeoxynucleotides showed a tumor-killing effect through TRAIL/Apo-2L signaling [280].

The role of hypoxia in B cells in a tumoral context is still under discussion. Lee and colleagues showed that the deletion of HIF-1α induced B cell infiltration and accelerated the progression of pancreatic cancer [206]. On the contrary, B regulatory cells are particularly important in cancer, as they exert an immunosuppressive role. B regulatory cells secrete TGFβ and IL-10; in fact, IL-10 suppresses innate immune responses, which results in tumoral protection [281]. However, in cancer, hypoxia seems to enhance the IL-10 production by B cells. For instance, hypoxia induces the expression of the high-mobility group B1 (HMGB1) in the tumor cell-released autophagosomes (TRAPs), which in turn induce IL-10 production in B cells, with a consequent suppression of T cell function and thus protection for tumors against immune response [207].

### 3.7. Endothelial Cells

Diverse stimuli such as proinflammatory cytokines and TNF-α in the circulation activate endothelial cells, increasing their permeability and inducing leukocyte adhesion. Endothelial cells under proinflammatory conditions induce the expression of E-selectin [282] and P-selectin overexpression [283] to promote the “tethering” and “rolling” of leukocytes through interaction between selectins and their PSGL-1 ligands [284]. Endothelial cells also release cytokines such as IL-8, which binds to CXCR1 and CXCR2 on neutrophils [285,286], and chemokines including CCL2 or MCP-1, which act through CCR4 and CCR2 on T lymphocytes and monocytes [287,288]. Leukocyte rolling is controlled by the expression of specific adhesion molecules on leukocytes and endothelial cell surfaces. The endothelial cells express the surface integrins ICAM-1 and VCAM-1, leading to the expression, spreading, and clustering of receptors such as VLA-4 and LFA-1 in the leukocytes, mediating the adhesion and transmigration of the leukocytes into the subendothelial spaces of the vessel wall [288,289,290,291,292].

The mechanisms of adhesion of cancer cells to endothelial cells and the transmigration through the endothelium are still under discussion. Tremblay and colleagues demonstrated that E-selectin induced by IL-1 is necessary for the adhesion and rolling of circulating colon neoplastic cells on endothelial cells and also is required by subsequent diapedesis [293]. The authors suggested that colon cancer cells bind at endothelial cells and induce an endothelial cell retraction and blebbing; thus, cancer cells can be engulfed in large vacuoles and transported within and through the endothelial cells [293]. In this sense, it has been demonstrated that breast adenocarcinoma MCF-7 cells were able to adhere to endothelial cells and promote their retraction as well as promote the apoptosis of HUVEC, inducing transendothelial migration [294,295]. In addition, Laferrière and colleagues reported that TNF-α mediated the adhesion between HT-29 colon cancer cells and HUVEC cells via E-selectin upregulation [296]. This process promotes the activation of SAPK2/p38/HSP27signaling in HT-29, enhancing mobility and transendothelial migration [282]. Furthermore, it has been suggested that tumor cells promote the apoptotic death of endothelial cells and migrate through a cavity formed in an endothelial cell monolayer [297].

In addition, it has been proposed that the tumor cell transmigrates through the endothelial cell–cell contacts [297]. In this sense, vascular tissues showed that VE-cadherin–containing adherent junctions were relocated and not opened or disrupted, whereas PECAM-1–containing junctions were opened during PMN transendothelial migration [298].

The permeability of endothelial cells to circulating tumor cells (CTCs) has a key role in the induction of metastasis, and this characteristic could be affected by hypoxia. Using a model of lung cancer, acute hypoxia leads to the stabilization of HIF-1α, increasing microvascular permeability and allowing the retention of myeloid cells, thus establishing a pro-metastatic environment characterized by a decrease in endothelial cells (CD31+CD45−). The expression of HIF-2α predominates in chronic hypoxia, decreasing the capacity of endothelial cells to permeate to CTC. In hypoxic conditions, the intercellular adhesion molecule 1 (ICAM1), involved in endothelium-macrophage adhesion, and the levels of CCL2, a cytokine that allows the interaction of endothelial cells and macrophages, were elevated, contributing to macrophage recruitment and metastasis. Moreover, myeloid cell infiltration was notably higher [208].

On the other hand, Schmedtje and colleagues reported that hypoxia induces the transcription of COX-2 through p65 NF-κB activation in HUVEC cells [299]. It has been suggested that prostaglandin and PAF synthesis in endothelial cells by COX-2 and PLA2 induces the adherence of neutrophils to the endothelium after hypoxia [209,300]. It has also been demonstrated that hypoxia promotes the endothelial ICAM-1 upregulation as well as monocyte–endothelium interaction by HIF1/Arg2/mitochondrial ROS [210].

## 4. Targeting Tumor Hypoxia and Inflammation

The hypoxic phenomenon around the tumoral cells involves an intricate flux of immune cells, which ultimately facilitates tumoral growth. In addition, this characteristic is related to resistance to radio and chemotherapy. However, with the increasing knowledge of these interactions, new options are available for different therapeutic approaches.

The perspectives are encouraging, and new alternatives to regulate the actions of NF-κB and HIF have been tested in a model of colitis, a factor related to the early onset of colon cancer [301,302]. For example, two natural-origin compounds, caffeic acid phenethyl ester (CAPE) and piceatannol (PIC), were nano-encapsulated in an albumin matrix and reduced the inflammation and also the levels of HIF-1α and p65 in an in vivo model with mice [301], though the mechanism for this effect was not clear. In the near future, there will likely be more alternatives to manage inflammation and cancer.

Another option is to target HIF in the tumoral cells. There are several inhibitors at different levels of the HIF pathway, including the dimerization of HIF-1α/HIF-1β, the binding of HIF-1α to DNA, or the interactions of HIF-1α with other proteins. In addition, other inhibitors act indirectly, avoiding the HIF-1α translation and stabilization or, on the contrary, benefitting the degradation of HIF-1α [303]. Recently, a HIF-2α inhibitor called Belzutifan was approved by the FDA and used to treat tumors derived from VHL diseases such as renal cell carcinoma [304,305]. Patients with VHL disease will develop several tumors in their lifetime that require surgery; however, systemic therapy such as HIF-2α inhibitor administration in patients with lesions less than 3 cm in diameter would reduce their surgical interventions and the rate of metastasis. Nearly 50% of the patients with ccRCC showed an objective response, even though the expected secondary effects such as anemia or fatigue were observed. Moreover, an improvement in pancreatic lesions and hemangioblastoma was also documented. Therefore, the downregulation of hypoxic mediators such as HIF-2α results in a reduction of the proliferation rate of tumoral cells with VHL defects [306].

HCC is characterized by extensive vascularization. The overexpression of VHL protein as a therapeutic approach attempts to decrease the pro-tumorigenic effects of the HIF pathway. For instance, the overexpression of VHL with the coadministration of doxorubicin leads to a decrease in cell proliferation and angiogenesis, in addition to the downregulation of NF-κB, using a murine model [307]. Moreover, Iwamoto and colleagues analyzed the effect of a synthetic sulfoglycolipid known as sulfoquinovosyl-acylpropanediol (SQAP) to upregulate the expression of VHL protein using samples of HCC xenotransplanted to mice. SQAP decreased the expression of HIF-1α, HIF-2α, and NF-κB, leading to a downregulation of the tumoral angiogenesis and thus making VHL a possible therapeutic target in vascularized tumors [308].

Several strategies point to inhibiting the TGF-β effects, which are upregulated in hypoxia. For instance, the administration of an inhibitor of the TGFBR1, known as LY2109761, together with transarterial embolization (TAE) in a model of liver cancer resulted in suppression of tumor growth and metastasis [309]. Another approach attempted to inhibit TGF-β through a TGFBR2 blockade in CD4+ T cells to increase an antitumoral response in tumors resistant to antiangiogenic immunotherapies. To do this, the authors designed a CD4 TGF-β Trap (4T-Trap), which consisted of the TGF-β-neutralizing TGFBR2 extracellular domain attached to a non-immunosuppressive CD 4 antibody; 4T-Trap inhibited the TGF-β signaling cascade in TH cells, leading to remodeling of vasculature and cancer cell death [310].

A different approach that has gained interest is centered on TAMs. One method could be the inhibition of the recruitment of the macrophages to the tumor through the CCL2/CCR2 axis. This includes inhibitors such as trabectedin, monoclonal antibodies such as carlumab directed to CCL2, or inhibitors of CCR2 such as PF-04136309 [311]. The complementary therapy of TAMs as a target could help in synergy with other approaches. For example, an inhibitor of CXCL-12 known as Olaptesed pegol (NOX-A12) prevented the recruitment of the macrophages in a hypoxic environment and helped to prevent the resistance of an anti-angiogenic approach in a glioma model in vivo [312].

The M-CSF/M-CSFR axis is also a target in the regulation of the macrophage pro-tumoral activity, as it activates the M2 macrophage activity, using synthetic inhibitors as PLX6134 or PLX3397 [313]. It is feasible to think about the reprogramming of the macrophages to the M1 phenotype through TLR agonists or anti-CD40 antibodies [311,313].

In an in silico study, bioactive compounds derived from Annona muricata, a tropical plant with anti-inflammatory effects, potentially disrupted the interaction between TLR4 and its ligand and thus the activation of HIF-1α [314].

In an interesting approach, a targeted therapy was proposed using macrophages as vectors to the tumoral cells carrying genes that are activated by hypoxia-responsive promoters, resulting in a more specific treatment focus in the hypoxic tumoral and resistant cells [154]. It has also been postulated to use macrophages as carriers of nanoparticles that can be directed to the tumor, exploiting the capability of recruitment around the tumor and providing more specific delivery of the antineoplastic drug in a glioma model [315]. In a similar approach, macrophages could engulf and transport nanoshells of gold, be attracted to the hypoxic tumoral areas, and then be eliminated by photothermal ablation [316].

In parallel, Hayasi and colleagues evaluated the pharmacological activation of p53 using DS-5272, an inhibitor of the interaction between MDM2 and non-mutated p53. This effect is related to the fact that MDM2 is a ubiquitin ligase that sends p53 to proteasomal degradation. They found that DS-5272 is capable of inducing an inflammatory response in leukemia cells, including the up-regulation of PDL-L1, which suppresses the activity of NK and T cells. In accordance, the expression of PDL-L1 depended on HIF-1α. Thus, hypoxia and the induction of the HIF-1α/PD-L1 axis lead to evasion of the immune system and induce a resistant phenotype in cells treated with DS-5272 [317].

Hypoxia is also a factor that challenges the success of immunotherapy. Immune checkpoint blockade (ICB) using PD-1 inhibitors was proposed to treat cancer; however, some patients failed to respond. Interestingly, the hypoxic microenvironment contributes to the resistance to immunotherapy. In this sense, Kumagi and colleagues reported that high levels of lactate induced the expression of PD-1 in Treg cells. Glycolytic activity is associated with high lactate levels, and hypoxia could promote glycolysis; furthermore, Treg cells metabolize free fatty acids and can proliferate in hypoxic conditions. When lactate is introduced to the cell through the monocarboxylate transporter 1 (MCT1), NFAT1 is translocated to the nucleus to induce the expression of PD-1, which is linked to an immunosuppressive function and thus contributes to the tumoral evasion of the immune surveillance and the resistance to the therapy with ICB [318].

As the relation between the cells that integrate the TME is understood more deeply, new approaches can be proposed using different techniques, such as gene therapy. For example, a vector could be designed with a promoter constructed with hypoxic and cytokine-inducible enhancers targeting tumoral endothelial cells [319].

## 5. Conclusions

The intricate and complex TME is influenced by low levels of oxygen in solid tumors. An adaptation occurs to perpetuate the viability of tumoral cells and also affects the immune cellular components that enrich the TME. As we show in this review, inflammation and hypoxia are closely linked and support the progression of cancer. Particularly, hypoxia influences the cellular components of the inflammation response and becomes a factor in tumoral resistance.

Hopefully, having a more comprehensive panorama of the TME opens a new perspective to strategies that are directed not only to the tumoral but also surrounding immune cells to restrain the progression of cancer in a more integrative and effective manner.

## Figures and Tables

**Table 1:** Effects of hypoxia over cellular components of immune response.

Cell Type	Effects of Hypoxia	References
TAMs	Recruitment to hypoxic regions Release of pro-angiogenic factors and revascularization Promotion of secretion of MMPs in tumoral cells Switching to M2 phenotype	[154,155,156,157] [156,158,159,160] [161] [162,163,164,165,166]
CAFs	Induction and stabilization of pro-tumorigenic CAFs Reversal of pro-tumoral phenotype	[167,168,169,170,171] [172,173]
NK cells	Impairment of NK cells function	[174,175,176,177,178,179,180,181,182]
MDSC	Recruitment to tumor and inhibition of immune response Differentiation to TAMs Release of VEGF	[175,183,184,185,186,187] [188,189] [184,190,191,192]
T cells	Enhanced recruitment and lytic activity (CD8+) Decreased anti-tumoral functions of CD8+ Differentiation in TRM or exhausted T cells Reduction of antitumoral γδ T cells Differentiation of anti-inflammatory Tregs	[193,194,195,196,197] [198,199] [200,201,202] [203] [204,205]
B cells	Decrease of B cell infiltration Suppression of T cell antitumoral response	[206] [207]
Endothelial cells	Increase of microvascular permeability Increase of cell adhesion	[208] [209,210]

TAMs: Tumoral associated-macrophages; CAFs: cancer-associated fibroblasts; NKs, Natural Killers, MDSCs: Myeloid-Derived Suppressor Cells. See more details in the main text.

## Supplementary Materials

The following supporting information can be downloaded at: https://www.mdpi.com/article/10.3390/cancers14092291/s1, Figure S1: Connection between hypoxia and pyroptosis.
