# Supplementary figures and images for: Hypoxia as a Modulator of Inflammation and Immune Response in Cancer
Source: Cancers (Basel). 2022 May 4;14(9):2291. doi: 10.3390/cancers14092291 (PMC9099524; doi:10.3390/cancers14092291)

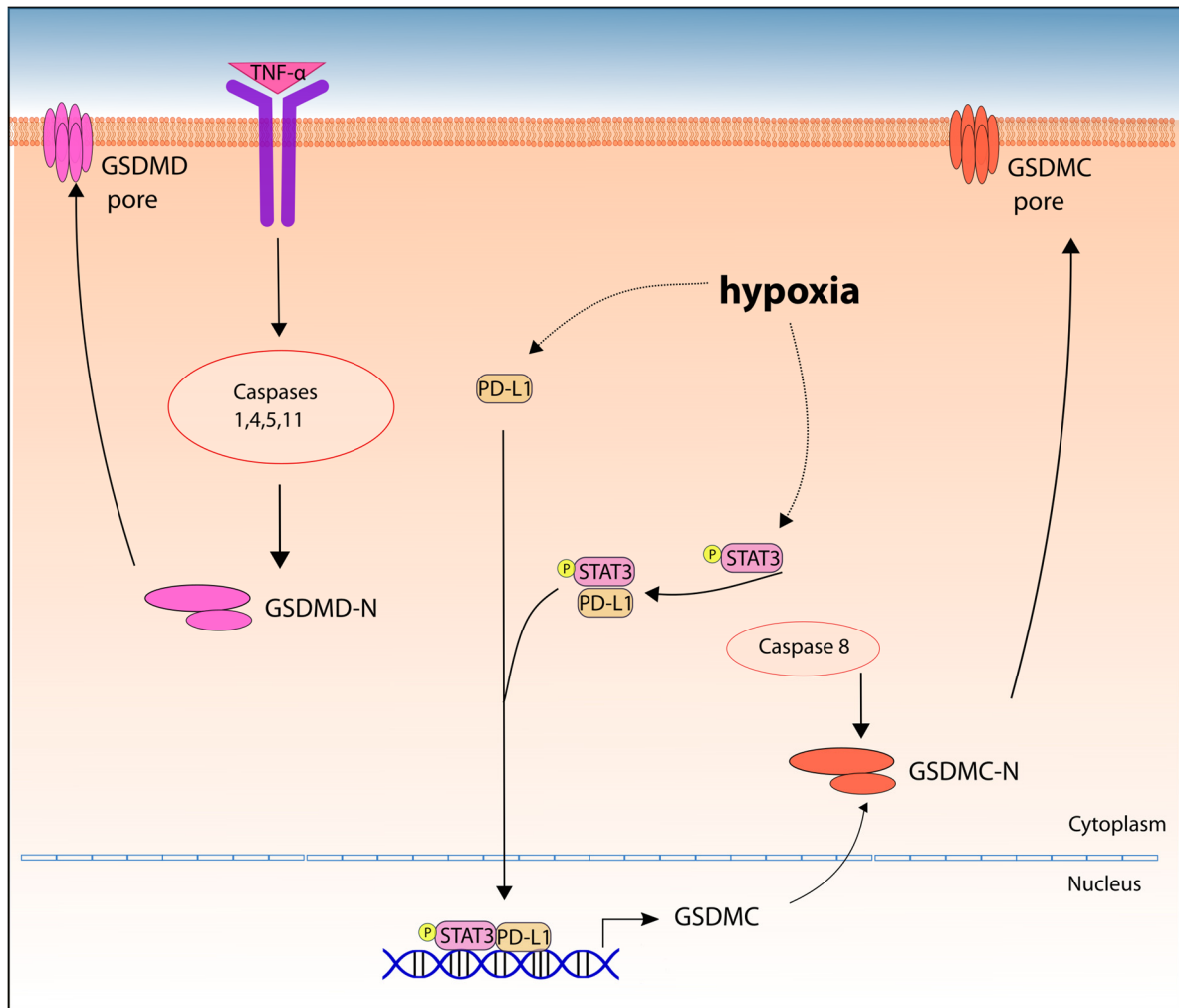

**Figure S1.** Connection between hypoxia and pyroptosis.

Supplement: Supplementary file 1 [file cancers-14-02291-s001.zip › cancers-1670589-supplementary.pdf]
